# Guanidinates as Alternative Ligands for Organometallic Complexes

**DOI:** 10.3390/molecules27185962

**Published:** 2022-09-13

**Authors:** Fernando Carrillo-Hermosilla, Rafael Fernández-Galán, Alberto Ramos, David Elorriaga

**Affiliations:** Departamento de Química Inorgánica, Orgánica y Bioquímica—Centro de Innovación en Química Avanzada (ORFEO−CINQA), Facultad de Ciencias y Tecnologías Químicas, Universidad de Castilla-La Mancha, 13071 Ciudad Real, Spain

**Keywords:** guanidine, organometallics, catalysis, materials

## Abstract

For decades, ligands such as phosphanes or cyclopentadienyl ring derivatives have dominated Coordination and Organometallic Chemistry. At the same time, alternative compounds have emerged that could compete either for a more practical and accessible synthesis or for greater control of steric and electronic properties. Guanidines, nitrogen-rich compounds, appear as one such potential alternatives as ligands or proligands. In addition to occurring in a plethora of natural compounds, and thus in compounds of pharmacological use, guanidines allow a wide variety of coordination modes to different metal centers along the periodic table, with their monoanionic chelate derivatives being the most common. In this review, we focused on the organometallic chemistry of guanidinato compounds, discussing selected examples of coordination modes, reactivity and uses in catalysis or materials science. We believe that these amazing ligands offer a new promise in Organometallic Chemistry.

## 1. Introduction

**Figure 1:**
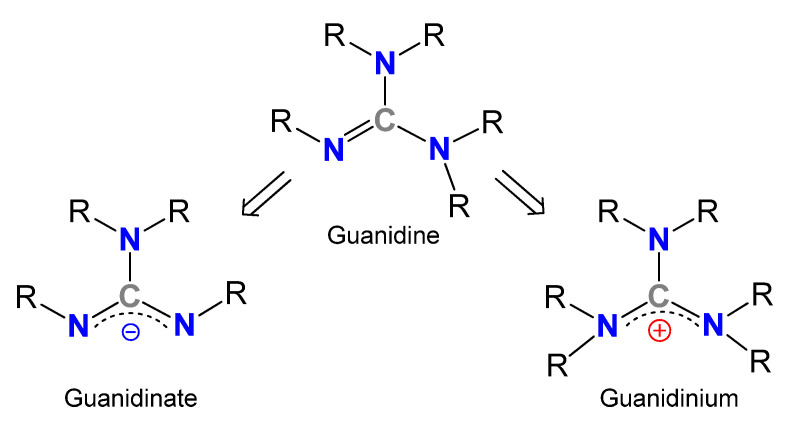
General structures of guanidines, guanidinates and guanidinium cations.

Guanidines, Y-shaped compounds of general formula R^1^N=C(NR^2^R^3^)(NR^4^R^5^) (R^1–5^=H, alkyl, aryl) (Figure 1), are very attractive organic molecules in different branches of Chemistry, exploiting their highly basic character [1,2] or the stability of their guanidinium cations, in which the positive charge could be delocalized over the three nitrogen atoms, and which are involved in many biological processes. For example, several enzymes containing the amino acid arginine take advantage of the presence of the side chain functional group guanidinium for the fixation of diverse substrates [3,4,5,6,7,8,9]. Similarly, the facility to form very stable hydrogen bonds allows the use of guanidines as organocatalysts in reactions, such as Diels–Alder reactions, addition reactions, CO_2_ capture and transformation to valuable products or the ring-opening polymerizations of lactones and lactides, even with the adequate control of the enantioselectivity [10,11,12,13,14,15,16,17,18,19,20,21,22,23,24,25,26,27,28,29,30,31,32]. It should be noted that the expected donor character of these derivatives and their anionic forms, the guanidinates, has led to a new and growing class of metal complexes. Although there are a significant number of examples where guanidines act as neutral ligands, the monoanionic forms exceed neutral or dianionic forms in examples, the *N*,*N’*-chelating being by far the most widely encountered coordination mode.

**Figure 2:**
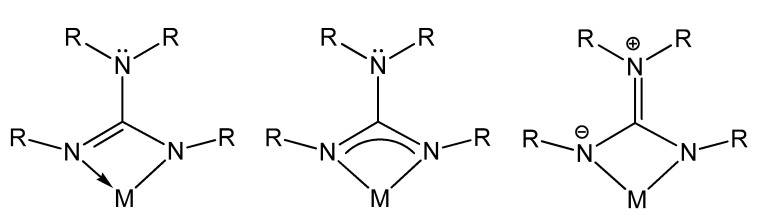
Electronic delocalization in guanidinato ligands.

The main feature of these chelating monoanionic guanidinato ligands is the electronic flexibility in coordination with metal centers due to the large electronic delocalization through its structure (Figure 2). This in turn provides stabilization to a wide variety of metal centers throughout the periodic table, in different oxidation states.

It is noteworthy that the great interest in the study of these compounds provides a broad palette of synthetic methods, involving both stoichiometric and catalytic processes [33,34,35,36,37].

Since metallic compounds based on these ligands were first reported in 1970 [38], they have shown great promise for applications in catalysis and materials science. In particular, guanidinato ligands have been investigated as an interesting alternative to classical ligands, such as cyclopentadienyl. An appropriate modification of the steric properties of these ligands can also play an important role in the commented applications of these complexes.

Although the chemistry of guanidinato derivatives (and their amidinato analogues) extends well beyond Organometallic Chemistry and has been covered in excellent reviews [39,40,41,42,43,44,45,46], this work is mainly concerned with systematically and comprehensively reviewing the examples of organometallic complexes, excluding some carbonyl derivatives, highlighting synthetic or structural aspects and the influence of the presence of these guanidinato ligands on their properties. The results reviewed here show a trend towards the search for targeted guanidines with the main goal of obtaining organometallic complexes with a use as potential homogeneous catalysts or as molecular precursors of solid materials.

## 2. Organometallic Chemistry of Guanidines

### 2.1. Main Group Complexes

Although, as mentioned above, the most common coordination mode of guanidinato ligands is the chelating k^2^-*N*,*N’* form, in the case of bulky lithium guanidinates, additional metal–carbon bond interactions can occur due to the presence of aromatic groups. These M-arene interactions have also been found for other group 1 elements, which are used to obtain guanidinates of other metals, with the purpose of generating steric protection for these metal centers.

**Figure 3:**
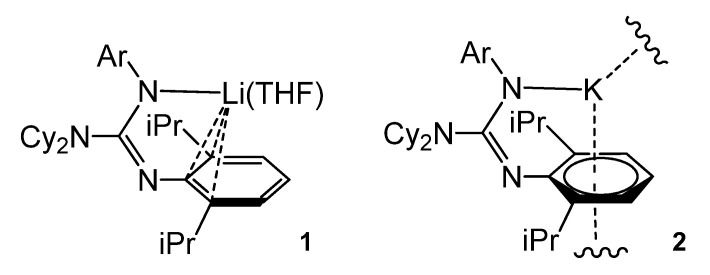
Lithium and potassium compounds with η^6^-arene coordination.

The deprotonation of the bulky guanidine ArNC(NCy_2_)N(H)Ar (Ar = 2,6-iPr_2_C_6_H_3_) with Bu*^n^*Li in THF led to the monomeric complex **1**. Similarly, the deprotonation of ArNC(NiPr_2_)N(H)Ar with K[N(SiMe_3_)_2_] gave an unsolvated polymer, **2**. whereas the arene-K interactions were close to η^6^- and the lithium compound showed an organometallic bonding of the type η^3^- (Figure 3) [47,48].

**Figure 4:**
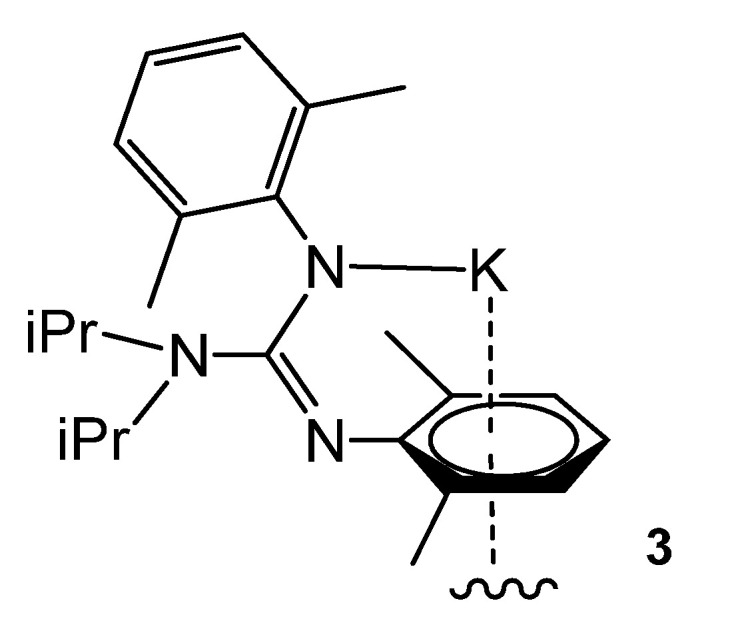
Polymeric potassium compound through η^6^-arene interactions.

The polymeric structure is repeated in derivative **3** with methyl groups on the aromatic rings. The solid-state structures reveal that the guanidinato ligand in these compounds retains the typical Z-anti configuration occurring in this class of bulky guanidines (Figure 4).

**Figure 5:**
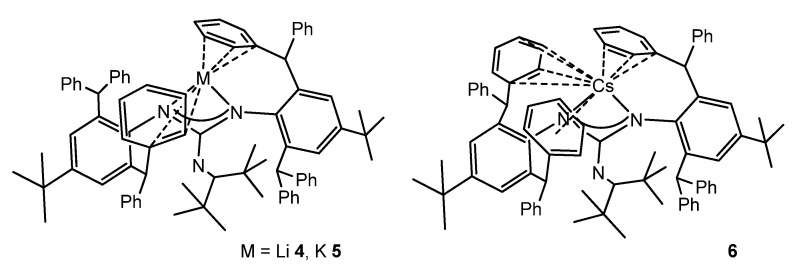
Alkali metal complexes exhibiting bond interactions with the aromatic substituents of the guanidinato ligands.

The reaction of bulky carbodiimide (ArN)_2_C (Ar = 2,6-(diphenylmethyl)-4-tert-butylphenyl) with LiNCtBu_2_ in tetrahydrofuran gives the monomeric lithium guanidinato compound **4**. Protonation and subsequent metalation allow the analogous compounds with K **5** and Cs **6** to be obtained (Figure 5). The solid-state structures of these complexes show that the alkali cations sit within a cavity formed from the peripheral phenyl rings of the diphenylmethyl substituents, which also provide additional support to the cations via intramolecular metal–arene interactions [49].

**Figure 6:**
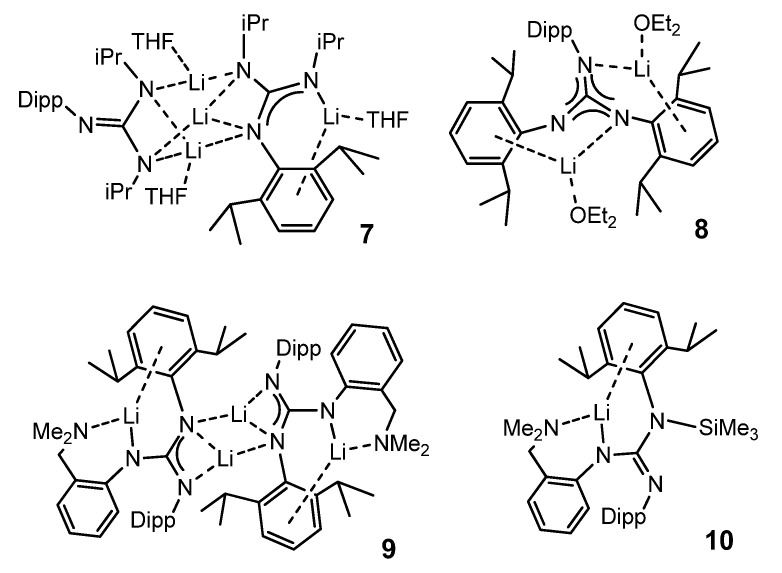
Alkali metal complexes exhibiting bond interactions with the aromatic substituents of the guanidinato ligands.

Other bulky guanidines, bearing 2,6-diisopropylphenyl (Dipp) group as substituent show, again, a coordination of the aromatic group to the metal center. This is the case in compounds **7**–**10** (Figure 6) [50].

**Figure 7:**
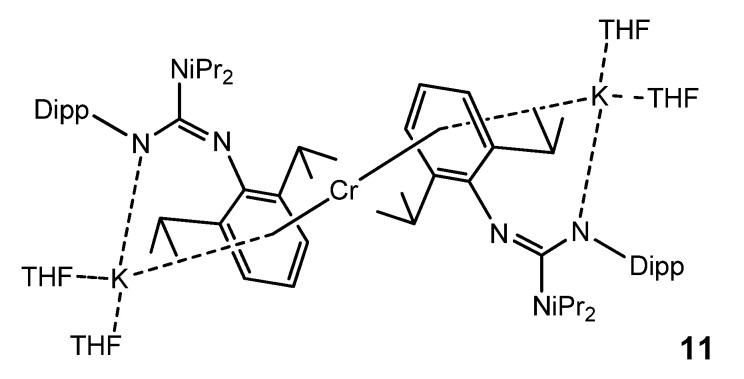
Monomeric chromium (0) bimetallic complex.

In the search for bimetallic systems with multiple metal-to-metal bonds, the reduction of a chromium guanidinate with KC_8_ led to a monomeric chromium (0) complex **11,** in which this metal atom is sandwiched between two arene units of two guanidinato ligands. The coordination of the arene units is not limited to the chromium atom, but the same aromatic substituents also coordinate one potassium cation in a similar fashion. Furthermore, the guanidinato ligands in **11** act as amides coordinating the K atom (Figure 7) [51].

**Figure 8:**
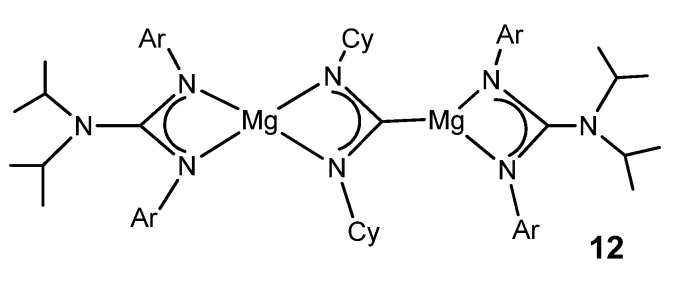
Magnesium magnesioamidinato complex.

The organometallic derivatives of most of the group 2 elements have been described. Very bulky guanidinato ligands enable the stabilization of abnormally low oxidation states for some metals. A dimeric magnesium(I) guanidinato complex acts as a facile and selective two-center/two-electron reductant towards a series of unsaturated substrates. The reaction with CyNCNCy gives the doubly reduced product **12** (Figure 8) [52].

**Figure 9:**
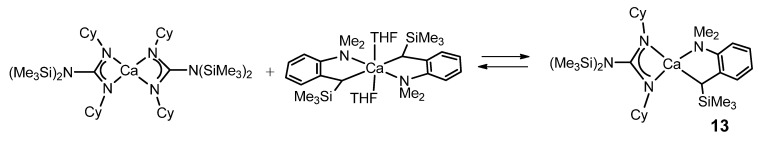
Heteroleptic benzylcalcium complex as the product of Schlenk equilibrium.

The Schlenk equilibrium between the bis-guanidinato complex of calcium [Ca{(Cy)N–C[N(SiMe_3_)_2_]–N(Cy)}_2_**·**(Et_2_O)] and the benzyl derivative [Ca{α-(Me_3_Si)-o-(Me_2_N)benzyl}_2_·(THF)_2_] yields mainly the heteroleptic benzylcalcium complex **13** (Figure 9) [53]. This complex, like many others that will appear throughout the text, is an example of one of the main applications of the compounds described in this work: to act as catalysts, in this case, in the catalytic polymerization of olefins in particular.

**Figure 10:**
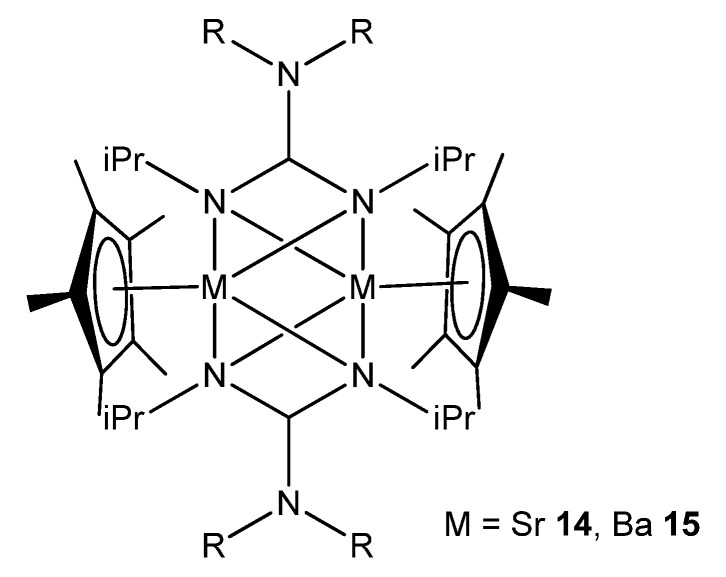
Dinuclear strontium and barium compounds with guanidinato chelate and bridging ligands.

In the case of the heavier group 2 elements, finding organometallic derivatives with potential uses is often hampered by their low stability and tendency to form poorly soluble oligomeric species. This is a consequence of the large ionic radius of the strontium and barium metal centers. These problems can be addressed by employing suitable bulky ligands that limit this tendency to aggregate. For example, a combination of guanidinato ligands and Cp* (Cp*=C_5_Me_5_) allows dinuclear strontium and barium species to be obtained, where the guanidinato chelate ligands also act as bridges between the metal atoms (Figure 10) [54].

**Figure 11:**
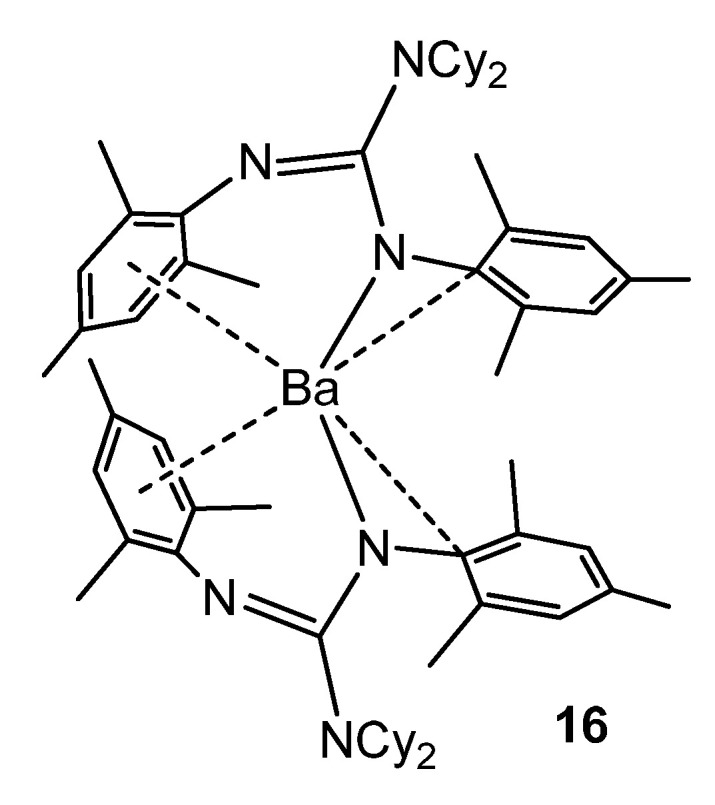
Electronic and steric stabilization of barium by aryl substituents in guanidinato ligands.

As with some group 1 elements, in the case of the barium complex **16**, electronic stabilization occurs, in addition to the steric protection of the guanidinato ligands, via interactions between the metal cation and the aromatic substituents of the ligands (Figure 11) [55].

**Figure 12:**
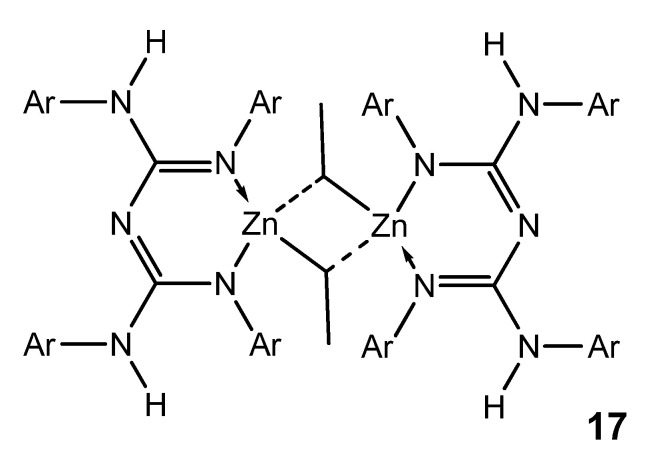
Bis-guanidianato alkyl zinc dimeric complex.

Although zinc is a d-block element, it is not considered a transition metal and is more appropriately considered as part of the main group. Interestingly, zinc organometallic guanidinates are scarce. The selectivity of the reaction between zinc dialkyls and guanidines is mediated by the identity of the guanidine substrate. The use of bis-guanidines with bulky aromatic substituents leads to the formation of dimer species, which have electron-deficient ethyl bridges common in the organometallic chemistry of the main group. These compounds are suitable precursors for obtaining stable zinc hydride derivatives, as they are active in the hydroboration and hydrosilylation of ketones (Figure 12) [56].

**Figure 13:**
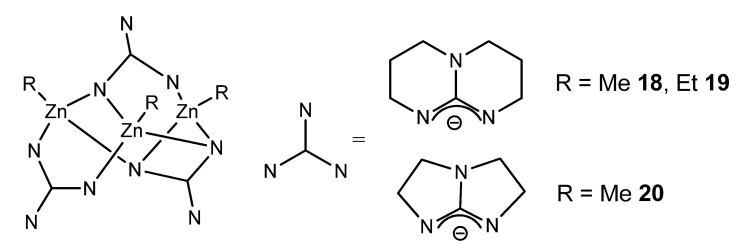
Bicyclic guanidinato complex acting as bridges in alkyl zinc complexes.

In the case of bicyclic guanidines, polynuclear systems can be achieved, where the more open projection of the orbitals of the nitrogen atoms of the amidine component of the ligand allows the formation of guanidinato bridges, as opposed to the formation of chelate systems (Figure 13) [57,58,59].

**Figure 14:**
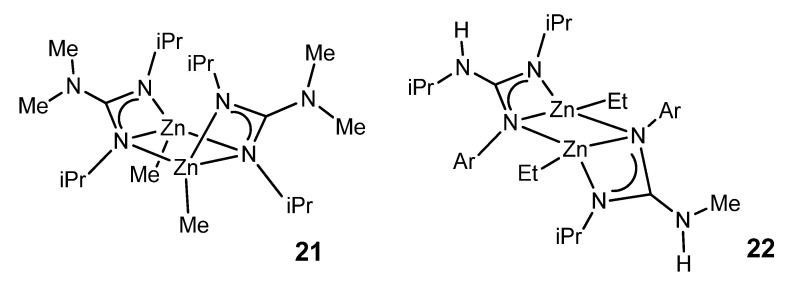
Guanidinato ligands acting both as chelate ligands and as a bridge between two zinc nuclei with boat and chair conformations.

With less sterically demanding guanidines, it is also possible to obtain dimer species in which the guanidinato ligands act both as chelate ligands and as a bridge between two zinc nuclei (Figure 14) [60,61]. The resistance to the protonation of the remaining alkyl group is manifested in the fact that protonation with a phenol involves the preferential cleavage of a Zn–N bond, generating a zinc complex with a neutral guanidine ligand coordinated by the imine nitrogen.

**Figure 15:**
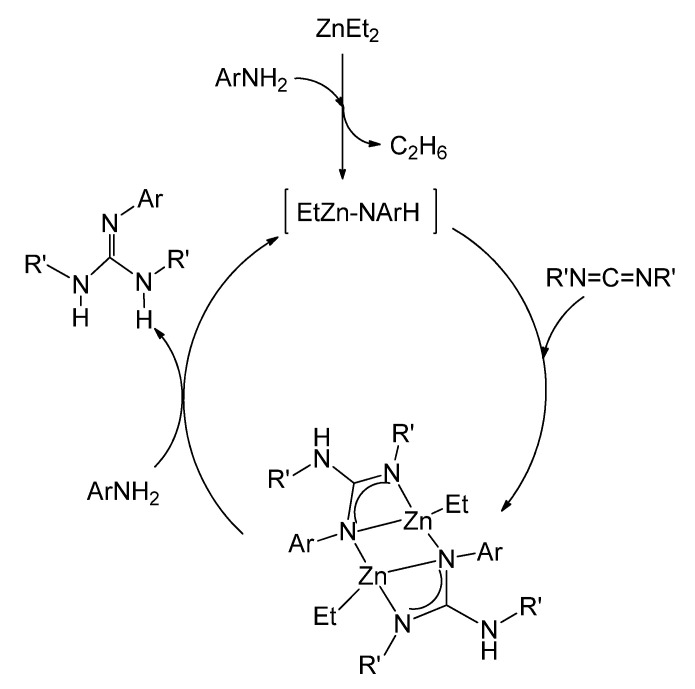
Catalytic guanylation of amines using ZnEt_2_ as a precatalyst.

The ethylzinc complexes of the type of **22**, with asymmetrically coordinated guanidinato ligands, have been shown to be intermediates for the catalytic production of new trisubstituted guanidines by the direct addition of anilines to carbodiimides (Figure 15) [61].

There is the widespread organometallic chemistry of aluminum compounds with guanidinate ligands. This interest can be seen in the trend of the last decades in the search for catalysts based on Main Group metals, looking for catalytic systems less expensive and with less impact on the environment than traditional catalysts based on transition metals [62].

Thus, coordination and organometallic aluminum complexes with a well-defined structure are among the most interesting due to their Lewis acidic characteristics and their availability.

**Figure 16:**
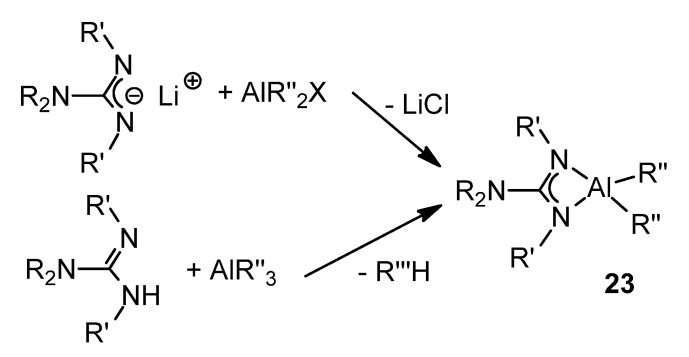
Synthesis of organoaluminum guanidinato complexes.

These aluminum guanidinate derivatives can be obtained both by metathesis of the ligand salt and an aluminum halide or by the protonolysis of alkylaluminums (Figure 16) [51,63,64,65,66,67]. 

**Figure 17:**
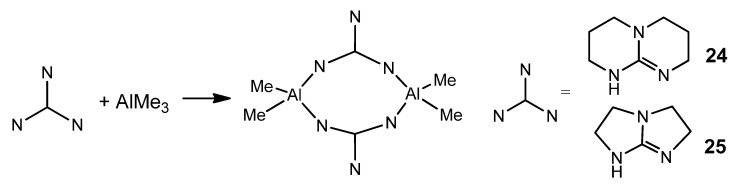
Bicyclic guanidinates acting as bridging ligands.

Depending on the type of guanidine, systems with chelating or bridging ligands can be obtained. As previously described for zinc [57,58,59], the arrangement of the electron pairs of the donor atoms in the bicyclic ligand makes a chelate-type coordination difficult (Figure 17).

An alternative is the migratory insertion of carbodiimides into AlR_2_(NR’_2_)-type compounds. In fact, it has been shown that the amido groups are preferred to the alkyl or halide groups for migration [68].

**Figure 18:**
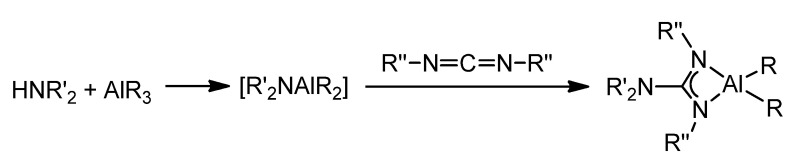
Insertion of carbodiimides into Al–N bonds of amido compounds.

These amido derivatives can be generated in situ and lead to the insertion of the carbodiimide to form the guanidinato species (Figure 18). It should be noted that this can occur during the catalytic process of addition of amines to carbodiimides to form new guanidines. The acid–base reaction between an aluminum alkyl and an N–H bond of the amine results in the formation of the amido species which, via nucleophilic addition to the carbodiimide, followed by protonolysis by another amine molecule of the guanidinato intermediate, gives rise to the corresponding guanidine [64,69,70].

**Figure 19:**
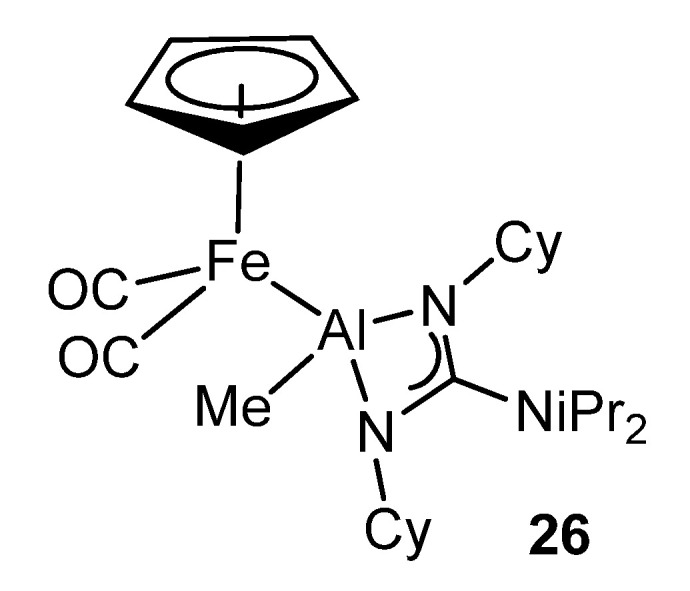
Bimetallic iron–aluminum compound.

The high kinetic inertness and thermodynamic stability provided by the guanidinato ligands to their complexes makes them very interesting precursors for the formation of transition metal–aluminum bonds by suitable reactions with organometallic anions (Figure 19) [71].

**Figure 20:**
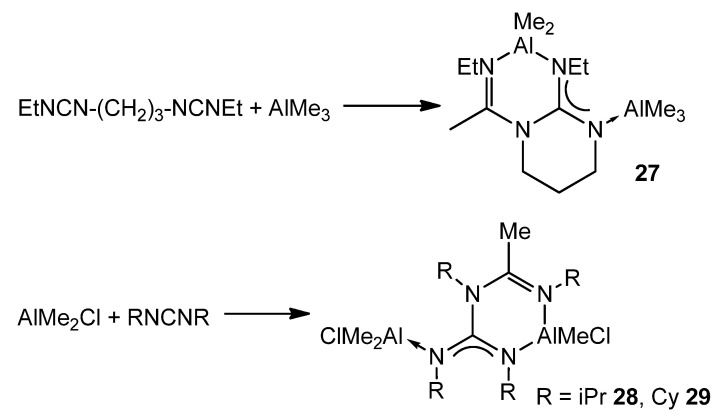
Double insertion of carbodiimides.

An interesting example of how a guanidinato ligand is obtained occurs when alkylaluminums and a biscarbodiimide, or two carbodiimide molecules, react at high temperatures. Instead of the desired amidinate, after the insertion process of one of the carbodiimide groups, the reaction proceeds with the attack of the nitrogen of the formed amidinate to the electrophilic carbon atom of the second carbodiimide (Figure 20) [72,73].

This reaction has also been observed for aluminum guanidinato compounds but depends on the nature of the substituents on the carbodiimide. Thus, [Al{k^2^-(NCy)(NPh)C(Net_2_)}Me_2_] reacts with CyN=C=NPh, but not with CyN=C=Ncy, to give rise to a complex similar to those of Figure 20 [74].

The potential of some of these organometallic complexes to act as catalysts is manifested in their ability to selectively reduce the carbonyl group of unsaturated aldehydes and ketones by the Meerwein–Ponndorf–Verley reaction [67,75].

**Figure 21:**
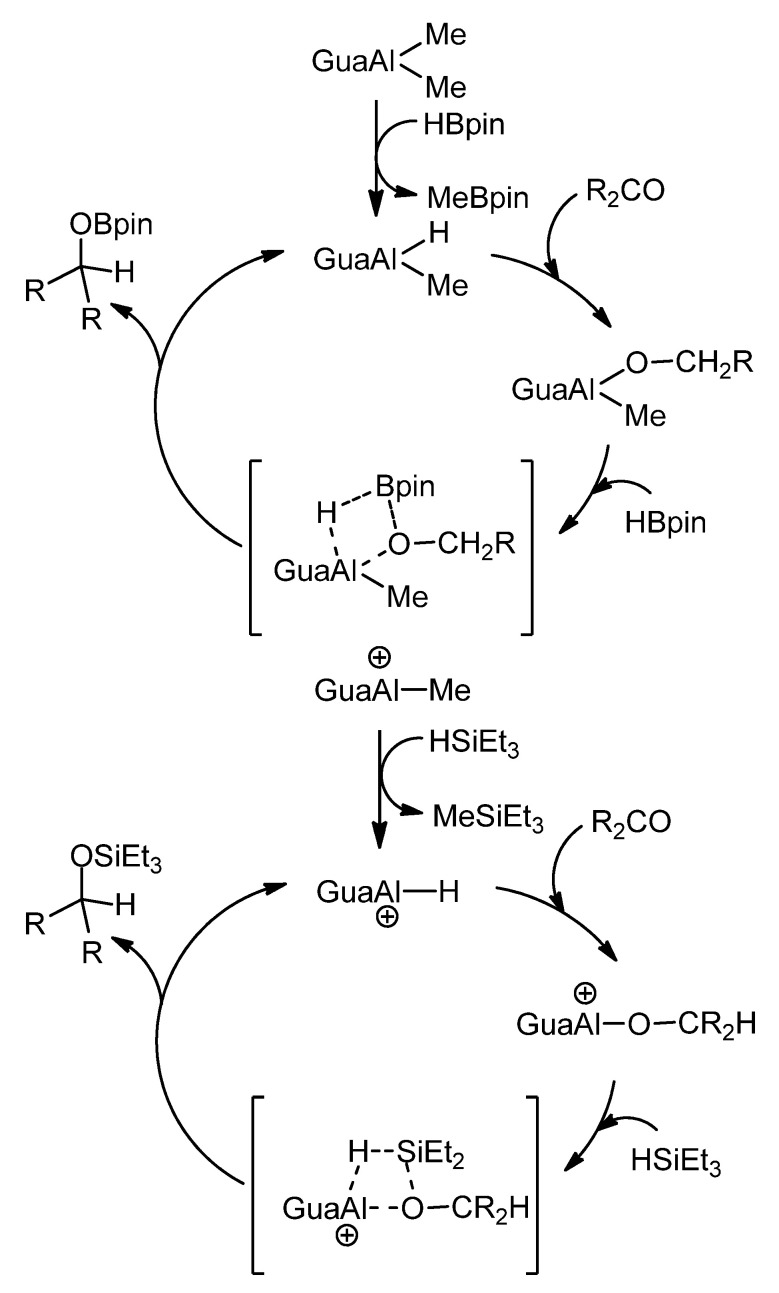
Hydroboration and hydrosilylation of ketones by aluminum hydride compounds.

In relation to ketone and aldehyde reduction, the feasibility of transforming some of these organometallic complexes into hydride derivatives should be highlighted. From bis-guanidines, mononuclear and dinuclear hydroboration or hydrosilylation catalysts can be obtained (Figure 21) [76,77].

**Figure 22:**
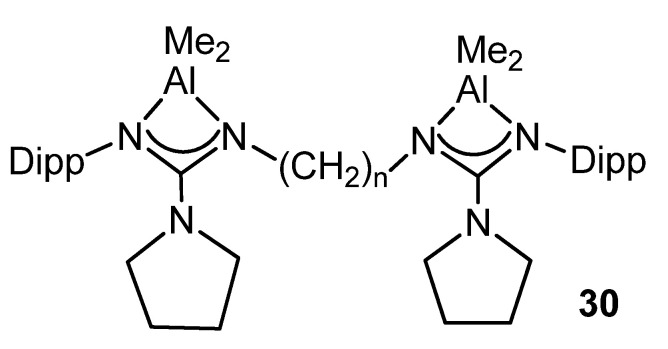
Dinuclear aluminum compounds, catalyst for ring-opening polymerization (*n* = 2, 3, 4).

Likewise, the dinuclear complexes of the type **30** (Figure 22) are efficient catalysts for the ring-opening polymerization of L-lactide and ε-caprolactone [78].

**Figure 23:**
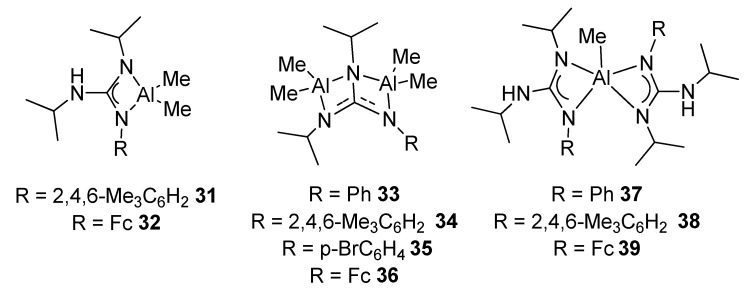
Mononuclear and dinuclear aluminum compounds with different coordination numbers.

The protonolysis of AlMe_3_ with trisubstituted arylguanidines allows to prepare mono- and dinuclear complexes, with different coordination modes. Through an appropriate choice of molar ratio and ligand structure, it is possible to achieve mononuclear complexes with the conventional chelate tetrahedral coordination, complexes with two AlMe_2_ moieties, or mononuclear with a rare pentacoordinated environment around the aluminum metal center (Figure 23). Some of these complexes are very active catalysts for the transformation of styrene oxide and CO_2_ into the corresponding cyclic carbonate [79].

**Figure 24:**
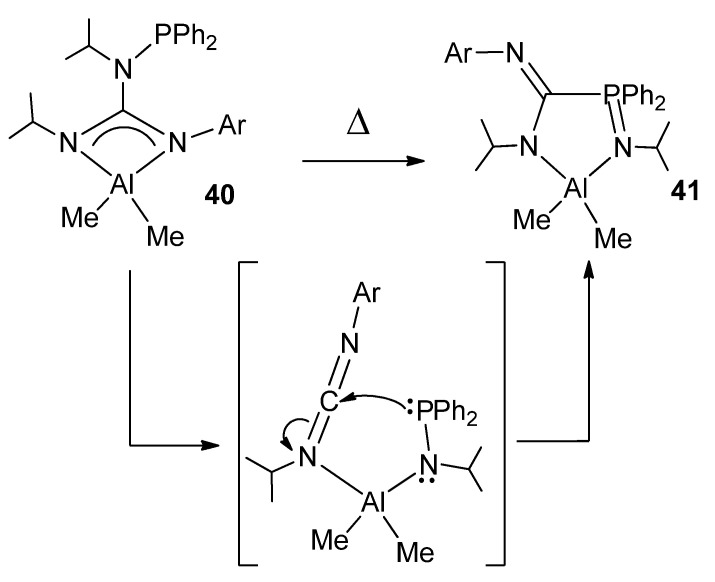
Rearrangement in phosphanoguanidinato derivatives.

The modification of guanidines with phosphane groups generates *N*-phosphanoguanidines, of general formula (HNR)(Ph_2_PNR)C(NAr). In the reaction with trimethylaluminum, phosphanoguanidinato derivatives were detected or isolated as the kinetic products of the reaction. Surprisingly, when the solutions of these organometallic complexes are heated, it results, selectively and quantitatively, in phosphanimine–amidinato compounds as the thermodynamic products of the process (Figure 24). A similar process was observed for other metals, such as Zn, Mg and Li [80,81].

Finally, as will be described later for other elements, because of its stability and volatility, organometallic aluminum guanidinates are potential precursors for the deposition of high-quality aluminum oxide by atomic layer deposition (ALD) [82].

Much less common are references to organometallic complexes of the other group 13 elements and other main group metals. These complexes of gallium, indium, thallium, tin and lead often display guanidinato ligands with substituents that complete the coordination sphere of the metal, such as aryl, amido, alkoxy or iminoacyl groups [83,84,85,86,87,88,89,90,91,92,93,94,95].

### 2.2. Transition Metal Complexes

**Figure 25:**
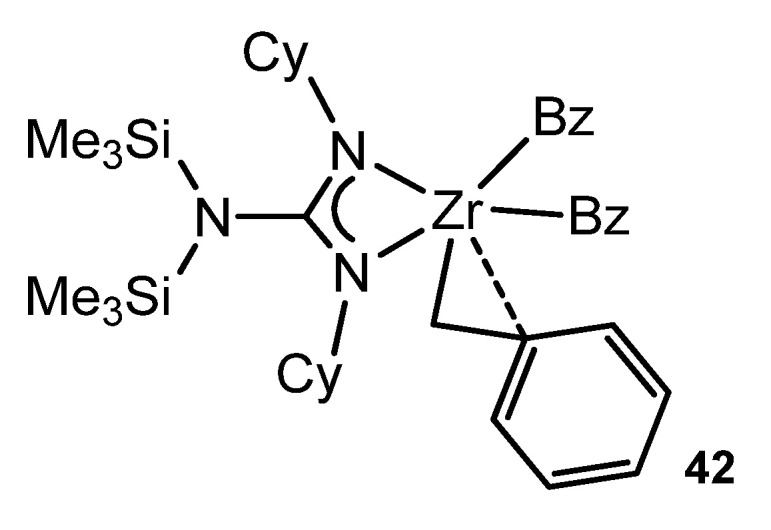
Zirconium complex with a η^2^-type bond interaction of a benzyl ligand.

Some of the alkali metal guanidinates described above were synthesized or prepared in situ as starting materials for reactions with transition metal halides and related precursors. Although the guanidinato derivatives of transition metals had already been described [38], Richeson’s group opened up the organometallic chemistry of metals of the early groups with this type of ligands. The alkylation of a symmetrically substituted guanidinato derivative of zirconium by metathesis reaction with PhCH_2_MgCl generates complex **42** (Figure 25). As with analogous classic complexes with cyclopentadienyl ligands, this compound exhibits a η^2^-type bond interaction of a benzyl ligand [96]. 

Alternatively, the same complex could be accessed via a toluene elimination reaction between neutral guanidine and tetrabenzylzirconium. This reaction can also be extended to other alkyltitanium derivatives [97,98,99].

There are far fewer examples of complexes containing a guanidinato (2−) ligand than the monoanionic ligands mentioned above [100,101]. For example, the reaction of [Cp*ZrCl_3_] with the dianion [{(PhN)_3_C}Li_2_] gave the zwitterionic species [Cp*Zr{C(NPh)_3_}Cl_2_Li(Et_2_O)(THF)], a complex active in ethylene polymerization [100]. As for their monoanionic analogues, these doubly negatively charged ligands also exhibit chelate-type coordination. Fluxional behavior was found in solution, and this involves a *syn/anti* isomerization process of the guanidinato (2−) ligand [101].

**Figure 26:**
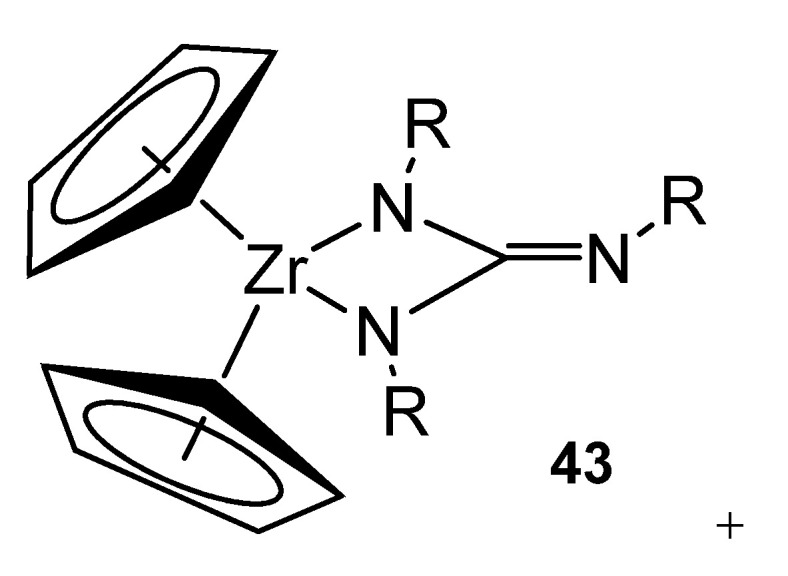
Dianionic zirconium guanidinato complex.

The formation of mixed cyclopentadienyl zirconium guanidinato derivatives can also be achieved by a formal [2 + 2] cycloaddition reaction of carbodiimides to imides. These azaallyl-like ligands still exhibit the κ^2^-chelate coordination described above and not the possible κ^3^-coordination similar to that of the allyl ligands, due to the orbitals involved in both cases (Figure 26). These complexes enable exchange reactions with other carbodiimides under mild conditions, which extends the family of this type of derivatives [102,103].

This type of complex still presents an uncoordinated imino group with a lone pair that could participate in the coordination to Lewis acid centers. However, the more than likely delocalization of these electrons along the ligand prevents such reactivity [104].

Whereas the alkylation with MeLi of the derivative Cp(C(C(NiPr)_2_(NMe_2_))ZrCl_2_ leads to a metathesis process to form the metallocene derivative and the bis-guanidinate, the use of the Grignard reagent MeMgCl or, more conveniently, the protonation reaction of the derivative (C_5_H_3_(SiMe_3_)_2_)ZrMe_3_ with triisopropylguanidine allows the obtention of dialkyl derivatives with guanidinato ligands [102,103,105].

**Figure 27:**
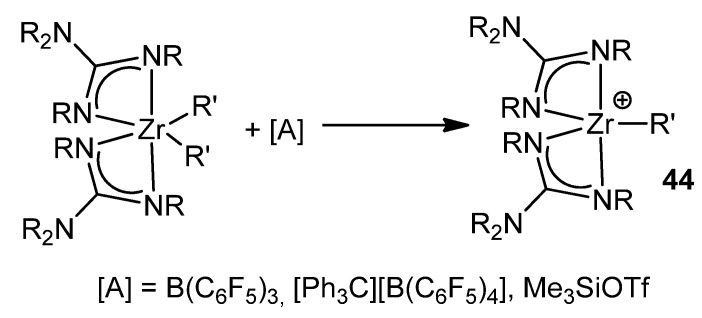
Cationic derivatives of a bis-guanidinato zirconium compound.

Following the similarity with the chemistry of metallocene derivatives, these neutral alkyl zirconium complexes with guanidinato ligands can be transformed into cationic derivatives by reaction with reagents, such as tris(pentafluorophenyl)borane, [Ph_3_C][B(C_6_F_5_]_3_]_4_ or Me_3_SiOTf (Figure 27).

**Figure 28:**
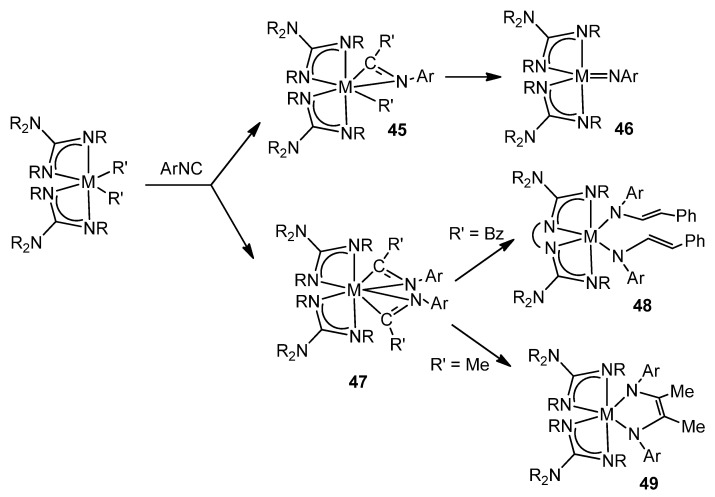
Aryl isonitriles insertion and the consecutive evolution of the iminoacyl groups.

The great influence of the geometry and the donor capacity of the guanidinato ligands on the reactivity of the complexes they form can be exemplified by studying the reactions of dialkyl derivatives with aryl isonitriles. The titanium or zirconium complexes of this class form intermediates, sometimes isolated, with η^2^-iminoacyl ligands for which subsequent transformations lead to vinylamido-type species, in the case of linked guanidinato ligands [106], while their unlinked analogues evolve towards imido or enediamido species (Figure 28) [107,108,109].

Occasionally, the lithiated guanidine derivative does not generate the expected chelate complex observed in most of the compounds described. Instead, in complexes such as Cp_2_TiCl(k^1^-N=C(NMe_2_)_2_) **50**, the guanidinate assumes an unusual binding mode, in the form of a monodentate ligand [110,111].

**Figure 29:**
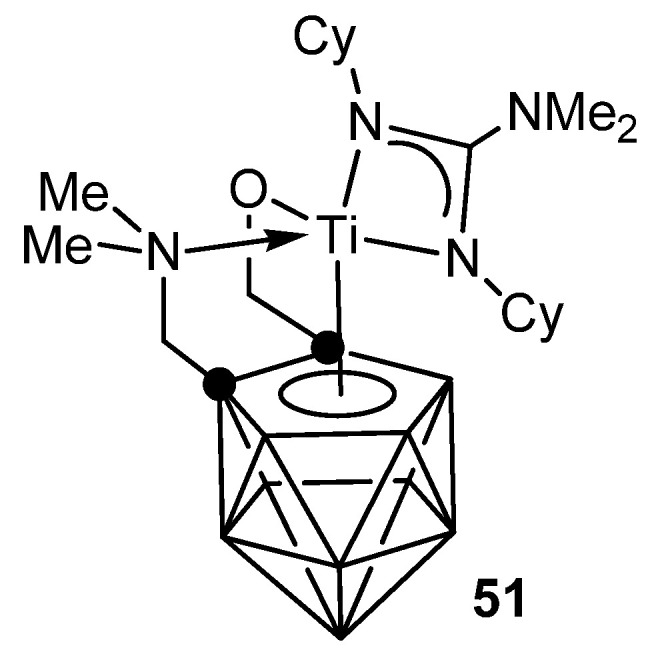
Metallocarborane complexes of titanium.

The metallacarborane complexes of zirconium and titanium can be obtained either by protonolysis with neutral guanidine or carbodiimide insertion in amido precursors. Titanium complex **51** is an effective catalyst for the addition of primary and secondary aliphatic and aromatic amines to carbodiimides with good functional group tolerance (Figure 29) [112,113].

**Figure 30:**
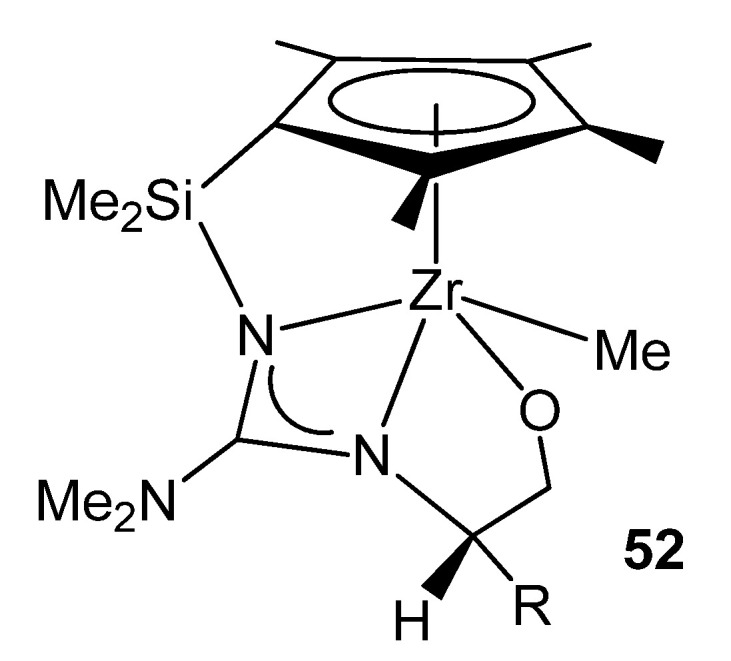
Chiral half-sandwich complexes with an oxazolinato group.

Using chiral modified 2-aminooxazolines gave rise to chiral half-sandwich complexes where the amino-oxazolinato group had undergone a ring opening and a migratory insertion of a dimethylamido ligand, providing a configurationally stable chiral-at-metal system (Figure 30) [114,115].

Although, as with metallocene derivatives, their application in catalytic processes makes the guanidinato complexes of group 4 metals very attractive compounds, these complexes have received interest as precursors to metal nitride or oxide thin films due to their nitrogen or carbon content, their potential to increase the volatility of the compound and their ability to stabilize the metal center due to their electronic flexibility. For example, thin films of carbonitrides or oxides of titanium, zirconium or hafnium can be obtained from guanidinato or mixed cyclopentadienyl guanidinato precursors. The above properties of these molecules satisfy the important requirements for their application in chemical deposition techniques, such as ALD or CVD [116,117,118,119].

**Figure 31:**
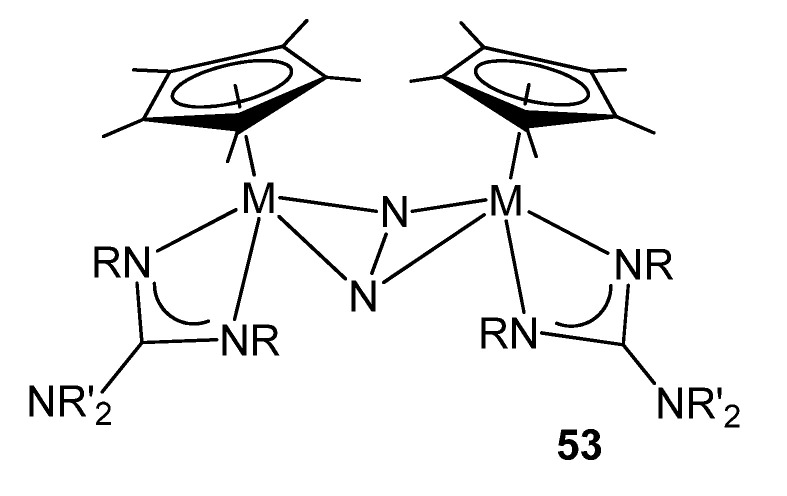
Coordination and activation of dinitrogen with group 4 guanidinato complexes.

As mentioned above, guanidinato ligands allow the stabilization of less common oxidation states for transition metals. Some mixed halide complexes of zirconium and hafnium can be reduced in the presence of N_2_ to form bimetallic complexes with a side-on-bridged dinitrogen molecule and proved to be very active toward both hydrosilylation and hydrogenation (Figure 31) [120].

**Figure 32:**
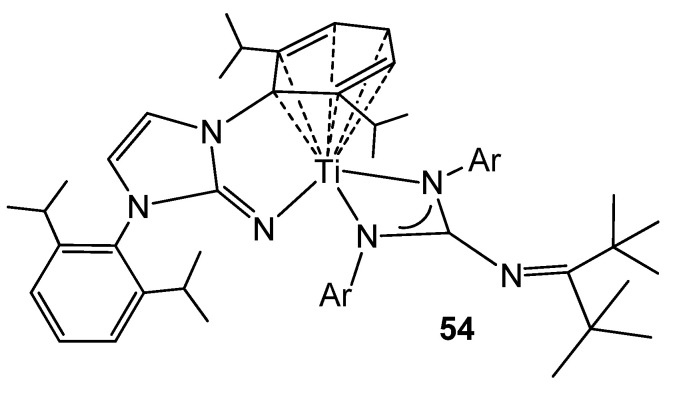
Arene–metal bond interaction in a titanium complex.

The titanium (II) complex **54** (Figure 32), obtained by a two-electron reduction from a dichloride precursor, exhibits an arene–M bond interaction similar to that previously described for group 1 elements, such as lithium or potassium. This type of complexes allows catalytic hydrogenation by hydrogen transfer to monocyclic and polycyclic arenes, mimicking the reactivity of late transition metals, as well as demonstrating a great ability to reductively activate a wide range of substrates, such as ketones, azides, N_2_O or C–F bonds [121,122,123].

**Figure 33:**
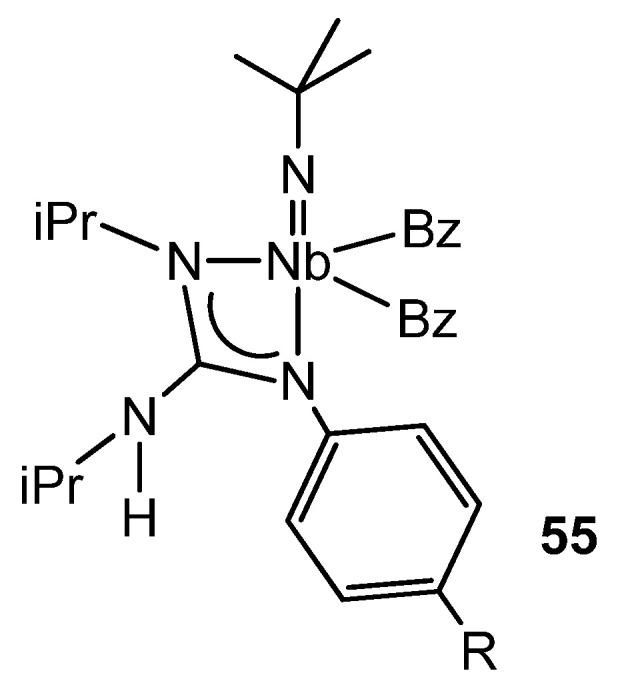
Asymmetric coordination of a guanidinato ligand in niobium complexes.

There is some parallelism between the organometallic chemistry of guanidinato complexes of groups 4 and 5. For example, the alkyl derivatives of niobium of the type **55** (Figure 33) show a η^2^-benzyl group in the solid state, and this is in rapid exchange with the other η^1^-benzyl ligand in solution. While the migratory insertion of tBuNC into the niobium–alkyl bonds gives rise to the corresponding bis(iminoacyl) derivatives, the insertion of an aryl isonitrile, XyNC (Xy = 2,6-Me_2_C_6_H_3_), into a niobium–benzyl bond results, as with some zirconium complexes [107], in the isomerization of an η^2^-iminoacyl group to a vinylamido-type one via a 1,2-hydrogen shift. The influence of the guanidinato ligand on this process is clear, since the same reaction does not take place starting from the precursor compound of the guanidinato complexes, the trialkylimido [NbBz_3_(NtBu)] (Bz = benzyl) [124,125].

These dibenzyl complexes with guanidinato ligands derived from diisopropylcarbodiimide and an aromatic amine show an asymmetric coordination of the ligand via an alkylamino nitrogen atom and the arylimino nitrogen atom. Computational studies confirm this preference, and the results suggest that electronic factors prevail over steric factors. In fact, when a guanidine with three alkyl substituents, one of them different, was used for the synthesis, a mixture of isomeric complexes was formed with the coordinated ligands without any selectivity for any of the nitrogen atoms. In addition, these complexes were proposed as intermediates in the mechanism of the catalytic guanylation of anilines, using the complex [NbBz_3_(NtBu)] as a precatalyst [126].

**Figure 34:**
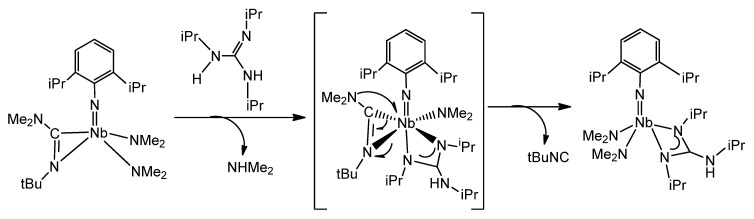
C–N activation mediated by a guanidine in an iminocarbamoyl compound.

Another example of the remarkable influence of the coordination of a guanidinato ligand on the reactivity on the coordination sphere of a metal is the process, in which an alkyl guanidine reacts with an iminocarbamoyl complex, which undergoes an easy cleavage of a C–N bond at room temperature (Figure 34) [127].

This process contrasts with that observed when migratory insertion of aryl isonitriles into the Ta-NMe_2_ bonds of a previously formed guanidinato complex was undertaken [128]. In this case, the iminocarbamoyl species [Ta(NMe_2_)[C(NiPr)_3_][η^2^-(Me_2_N)C=N(2,6-Me_2_C_6_H_3_)]_2_] **56**, which is stable, was formed.

As was the case for group 4 metals [120], guanidinato ligands stabilize organometallic tantalum species in a medium or low oxidation state [129,130,131]. For example, mixed cyclopentadienyl-guanidinato species of Ta(IV) give rise to N≡N bond cleavage to provide the bimetallic bis(μ-nitrido) complex, {Cp*Ta[N(iPr)C(NMe_2_)N(iPr)](μ-N)}_2_ **57**. The presence of the uncoordinated NMe_2_ group in the guanidinato ligand can serve to modulate the magnitude of the free energy barrier for N≡N bond cleavage.

Very recently, the participation of tantalum alkyl guanidinato derivatives of the type [Ta(hpp)_2_(CH_2_SiMe_3_)_3_] (hpp = 1,5,7-triazabicyclo [4.4.0]dec-5-ene) **58** has been studied as probable intermediates in the process of hydroaminoalkylation of terminal alkenes with a variety of secondary amine substrates to give substituted secondary amine products [132].

While the organometallic chemistry of group 6 derivatives is limited to a molybdenum complex, [(ArN)_2_MoMe{N(Cy)C[N(iPr)_2_]N(Cy)}] (Ar = 2,6-iPr_2_C_6_H_3_) **59** [133], and that of group 7 is non-existent, group 8 organometallic complexes with guanidine-derived ligands offer interesting structural and reactivity aspects.

**Figure 35:**
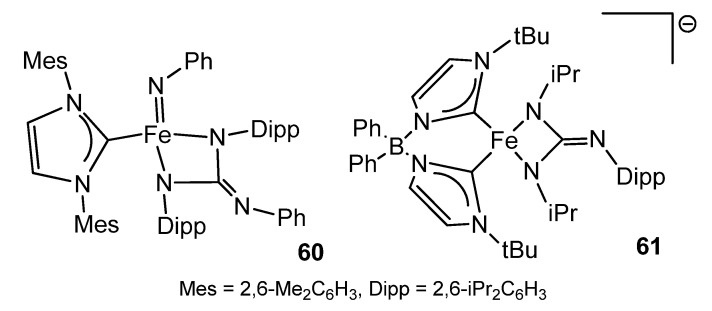
Iron dianionic guanidinato compounds.

Iron guanidinato (2−) complexes **60** and **61** (Figure 35) were obtained via a formal [2 + 2] cycloaddition of carbodiimide to imido precursors in high or low oxidation states [134,135].

**Figure 36:**
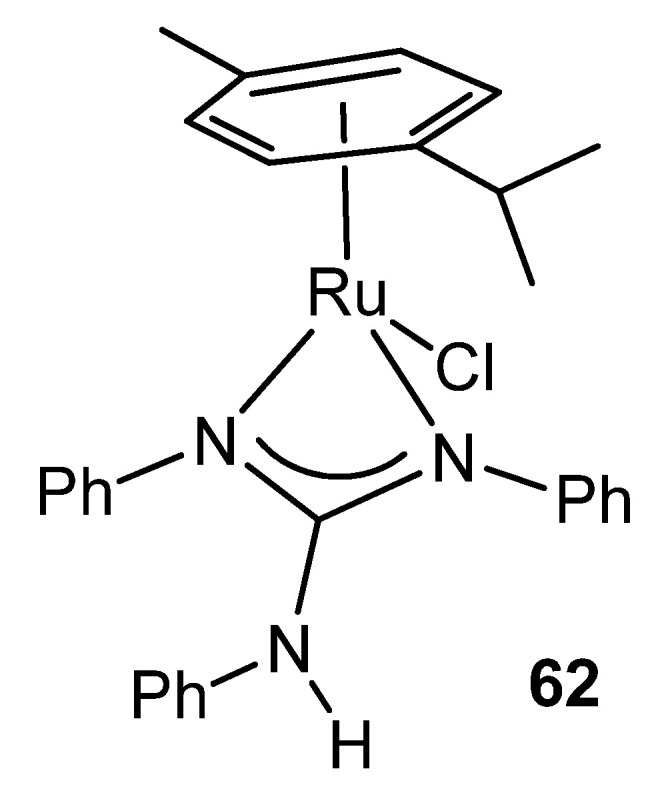
First example of a chelate guanidinato compound.

Ruthenium witnesses the first reference of a complex in which the guanidinato ligand acts as a chelate. Indeed, complex **62** contains an η^6^-bonded aromatic ligand, a terminal chloride and the chelating triphenylguanidine anion. The structure of this compound shows, for the first time, a feature that is repeated in many guanidinato complexes: the angles around the central carbon of the guanidine ligand total 360° indicating the strict planarity of the CN_3_ core (Figure 36) [136].

This coordination mode is present in other similar ruthenium complexes with both monoanionic and dianionic ligands [137,138,139,140,141].

**Figure 37:**
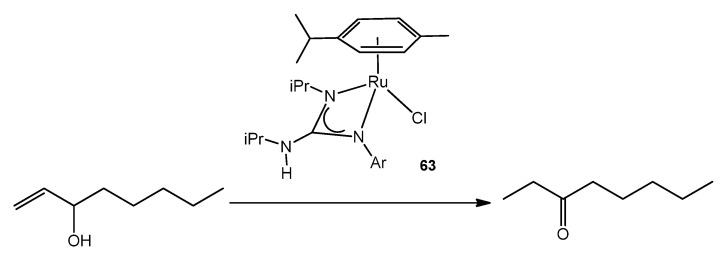
Ruthenium guanidinato complex acting as catalyst for allylic alcohol isomerization.

It is also worth noting that, despite the prominent role of ruthenium complexes in organic synthesis, there are only a few examples of guanidinato complexes reported to date that have been explored as catalysts. Complexes similar to those described are excellent catalysts for the redox isomerization of allylic alcohols in the absence of base (Figure 37) [142].

**Figure 38:**
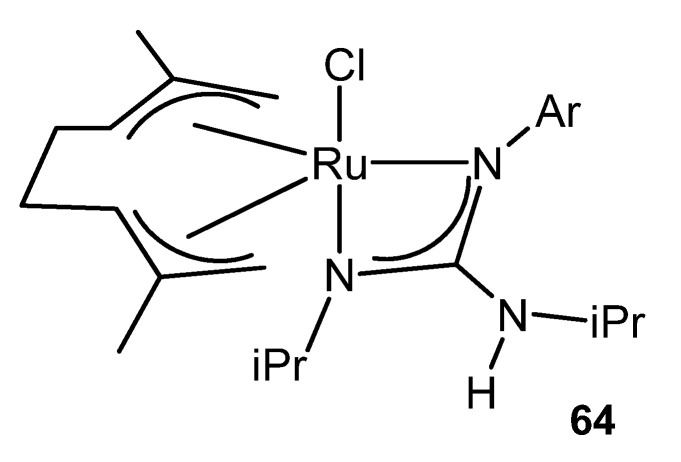
Ruthenium (IV) guanidinato complex [RuCl{κ^2^-C(NR)(NiPr)NHiPr}(η^3^:η^3^-C_10_H_16_)].

This catalytic process also takes place using ruthenium (IV) guanidinato complexes, namely [RuCl{κ^2^-C(NR)(NiPr)NHiPr}(η^3^:η^3^-C_10_H_16_)] **64** (Figure 38). This type of compounds are excellent precursors for the preparation of octahedral ruthenium (II) guanidinato complexes, *mer*-[RuCl{κ^2^-C(NR)(NiPr)-NHiPr}(CN-2,6-C_6_H_3_Me_2_)_3_] **65**, through the reductive elimination of the ligand 2,7-dimethylocta-2,6-diene-1,8-diyl with high stereoselectivity [143].

**Figure 39:**
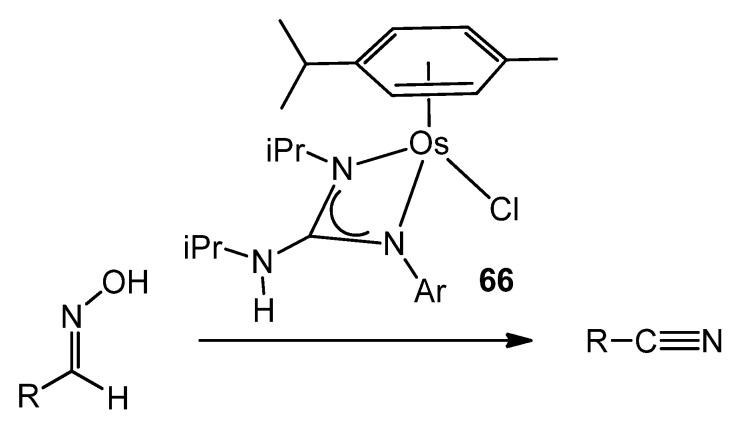
Osmium guanidinato complexes acting as aldoxime dehydration catalysts.

In contrast, less attention has been paid to osmium guanidinato complexes [137,144], although some examples were able to transform a wide variety of aldoximes selectively and catalytically into the corresponding nitriles (Figure 39).

**Figure 40:**
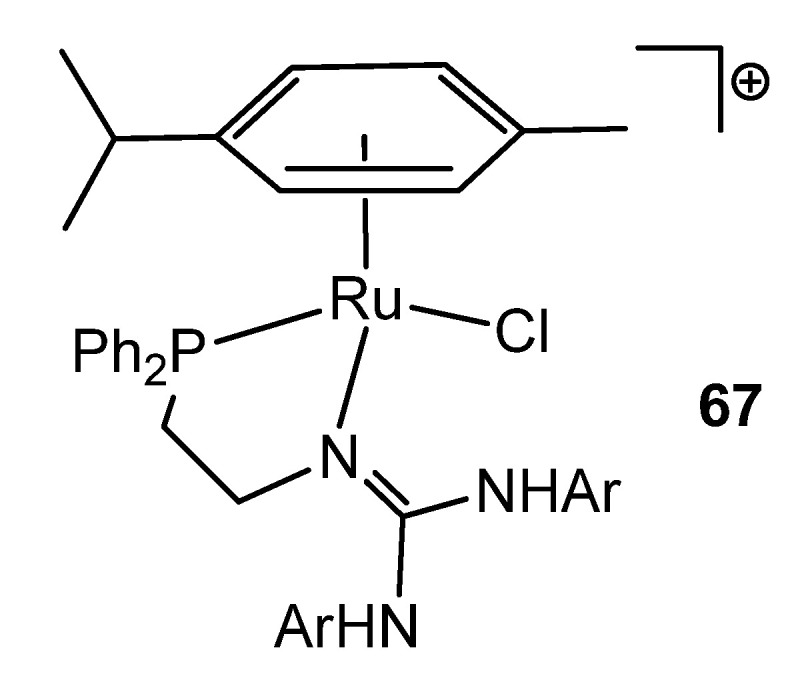
Phosphane-modified guanidine as ligand in a ruthenium complex.

Interestingly, in the search for new coordination modes, guanidines have been modified with other donor groups, such as phosphanes. In this way, ruthenium (II) (Figure 40) and osmium (II) complexes were obtained [145,146].

**Figure 41:**
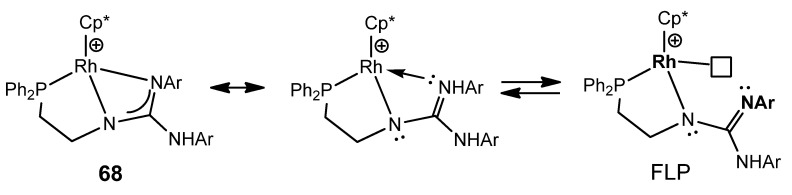
Frustrated Lewis pair based in rhodium phosphanoguanidinato complexes.

This type of multidentate ligand allows the preparation of the iridium and rhodium guanidinato complexes as **68**, which behave as a frustrated Lewis pair (FLP) capable of reversibly activating polar bonds, such as those of water, and non-polar bonds, such as those of dihydrogen (Figure 41) [147,148].

**Figure 42:**
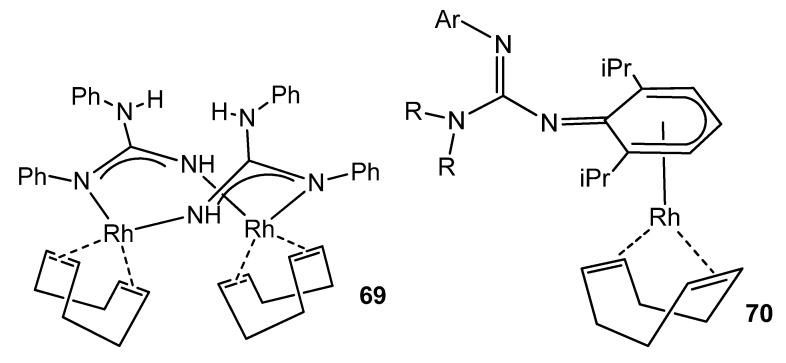
Bridging and arene coordination of guanidinato ligands in rhodium complexes.

Although the usual ligand arrangement in many rhodium complexes is the κ^2^-*N*,*N* chelate [137,149,150], examples are known where the guanidinate acts as a bridge between two metal centers [99], or even a complex where the anionic ligand coordinates the rhodium center in an unusual η^5^-cyclohexadienyl mode, before isomerizing to the conventional chelate form (Figure 42) [151].

**Figure 43:**
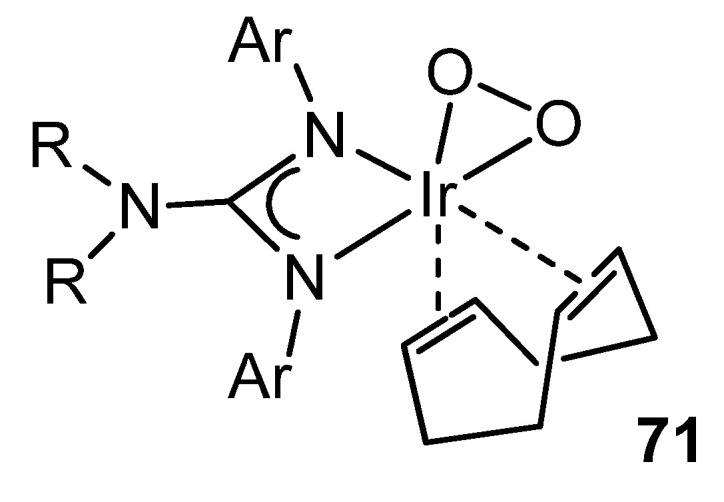
Dioxygen iridium complex.

Not surprisingly, as well as several examples of rhodium complexes with guanidinato ligands are known, the organometallic chemistry of analogous iridium compounds is wide. In addition to reactivities parallel to those described for rhodium [137,148,150], there are interesting examples of the influence of these ligands on the behavior of the metal. For example, the presence of the lone pair of electrons on the uncoordinated NR_2_ group increases the electron density of the ligand available for coordination to the metal. This obviously makes these ligands stronger donors than the related amidinato ligands. Consequently, if they coordinate to a metal in a low oxidation state, the possibility of reaching higher oxidation states increase. Thus, in the reactions of iridium guanidinato complexes with O_2_, the observed reactivity trends correlate well with both the electronic and steric properties of the substituents of the guanidinato ligands, allowing the stabilization and characterization of complexes with peroxo groups (Figure 43) [152,153,154,155].

This same donor capacity of the guanidinato ligand also lies at the basis of several catalytic processes involving organometallic iridium species, which allow the metathesis of carbodiimides and isocyanates, the carbodiimide cleavage or the cross-dimerization of terminal alkynes [156,157,158].

**Figure 44:**
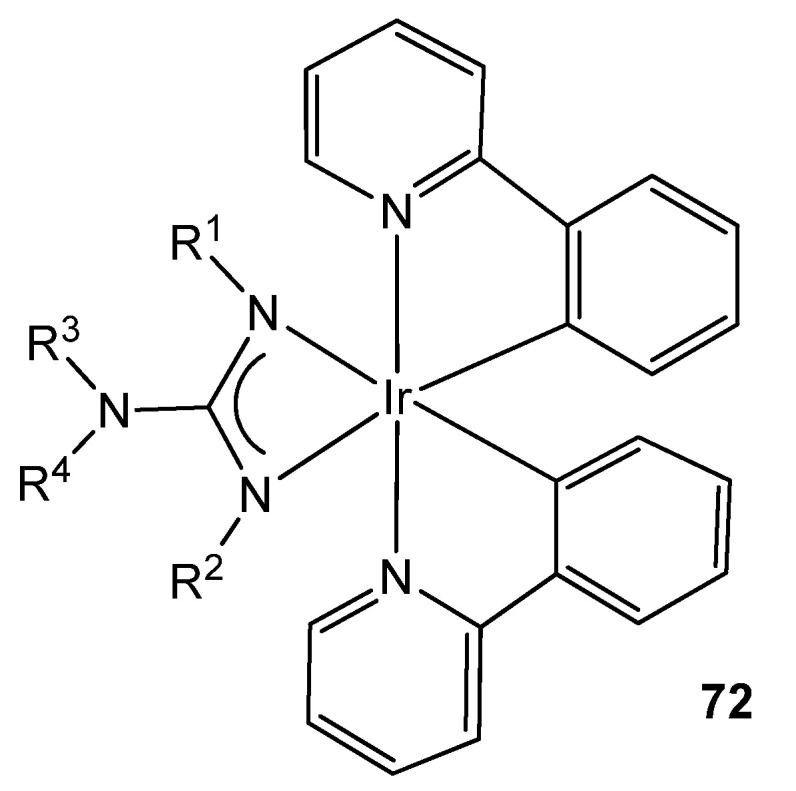
Luminescent iridium guanidinato compound.

Organometallic iridium (III) complexes are also useful as photophysical active materials in a wide variety of applications, e.g., as lighting devices, as dyes in solar panels, as bio-imaging agents or as photosensitizers for the production of hydrogen from water by photocatalysis [159]. It has been shown that these properties can be altered practically on a whim by modifying the ligands in these complexes. This is why, given the high tunability provided by the guanidinato ligands, they appear as excellent precursors of phosphorescent iridium cyclometalated complexes, becoming prominent in the preparation of Organic Light Emitting Devices (OLEDs), where the proper selection of ligand substituents allows high efficiencies and high variability of the emission color (Figure 44) [160,161,162,163].

The analogy between bulky guanidinates and β-diketiminates has been used to achieve the stabilization of Co(I) toluene adducts, as precursors of dimeric compounds with short Co–Co bonds [164].

**Figure 45:**
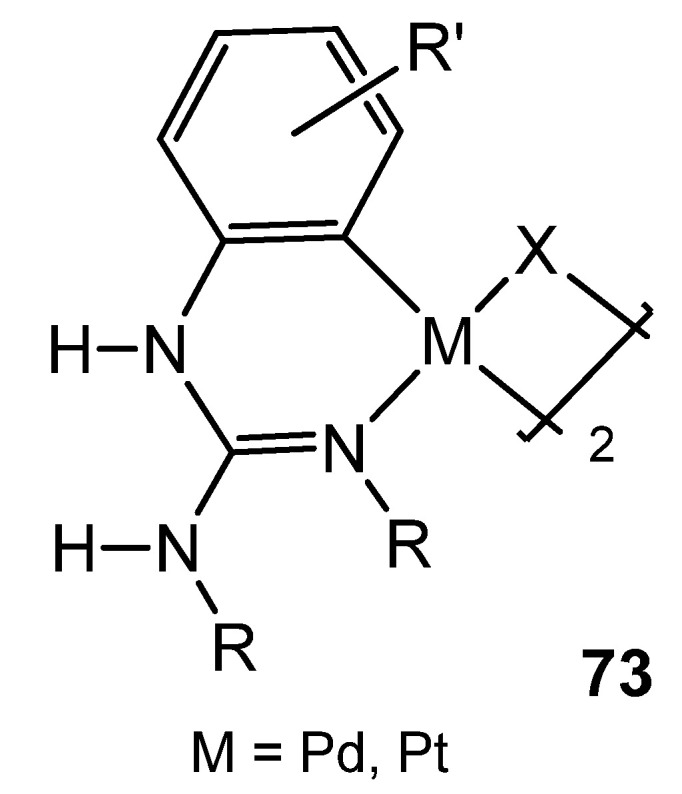
Cyclometalated guanidinato palladium and platinum complexes.

As with other platinum group metals, the first reference of group 10 organometallic complexes with guanidinato ligands presents species bearing dianionic ligands with chelate coordination [137,138,139,140]. Since then, it is worth noting the studies carried out by Thirupathi’s group, who have extensively studied aspects of the coordination chemistry of triarylguanidines towards palladium or platinum, in which the presence of Me or Ome substituents on the aromatic rings of the guanidines, together with their inherent basic properties, allowed the formation of interesting six-membered cyclometalated complexes, in which the guanidinato ligand shows a κ^2^-*C*,*N* coordination mode (Figure 45) [165,166,167,168,169,170,171,172,173,174,175].

**Figure 46:**
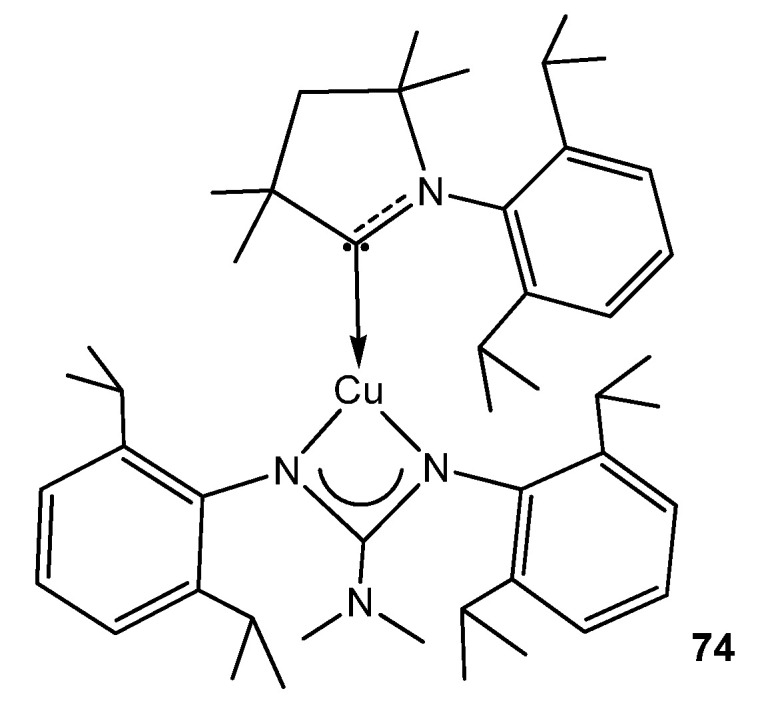
Phosphorescent carbene copper guanidinato compound.

To conclude this section, the organometallic derivatives of group 11 are limited to an example of a strongly phosphorescent copper (I) complex with an (alkyl)(amino)carbene ligand (Figure 46) [176].

### 2.3. Group 3 and the Lanthanoid Complexes

The group 3 elements and the lanthanoids represent a family of metals with very interesting properties in terms of chemical reactivity, luminescence or magnetism [177,178]. In particular, the guanidinato derivatives of these metals stand out for their potential use in catalytic processes or as molecular precursors of new ceramic materials. Although there are excellent reviews by Edelmann and Trifonov on rare earth amidinates and guanidinates [43,44,45], in this work we have selected the references focused on organometallic complexes, highlighting their usefulness and the possible differential nuances with the complexes of other groups of metals previously described.

**Figure 47:**
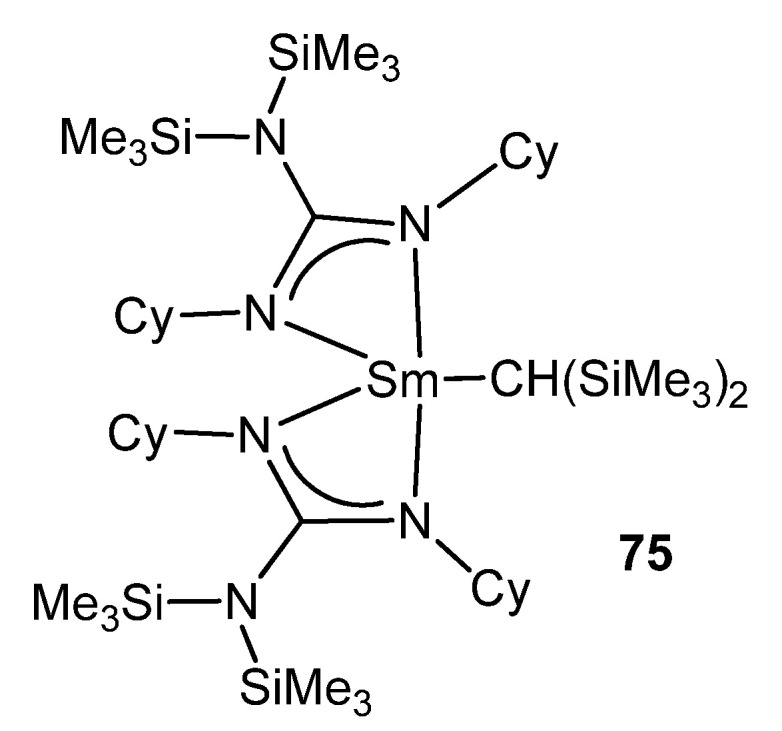
First reported organolanthanide complex with guanidinato ligands.

As with some of these examples, the metathesis of a lithium guanidinate and a metal halide is one of the main routes to rare-earth metal derivatives [179,180,181]. This is the case for **75**, the first reported organolanthanide complex supported by such a ligand [182]. After the formation of the halide derivative, the reaction with LiCH(SiMe_3_)_2_ yields a mononuclear organometallic compound (Figure 47).

Stable low-coordinate organometallic complexes, using bulky guanidinato ligands, allow agostic interactions between the yttrium atom and the tert-butyl group in the solid state for [{(Me_3_Si)_2_NC(NCy)_2_}_2_YtBu] **76** [183] or with two methyl carbon atoms of SiMe_3_ groups in [{(Me_3_Si)_2_NC(NCy)_2_}_2_Y(μ-CH_2_SiMe_3_)_2_Li] **77** [184].

Alternatively, the insertion reaction of carbodiimides into M–N bonds of amido derivatives allows a very efficient synthesis of organometallic complexes with symmetric and, mainly, asymmetric guanidinates [185,186].

**Figure 48:**
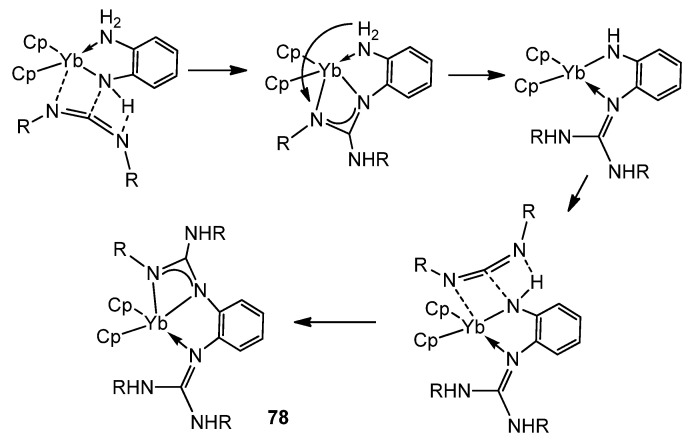
NH-assisted insertion of carbodiimides in amido ytterbium compounds.

The presence of NH groups on the ligands assists the insertion and can provide an interesting isomerization of these coordinated fragments (Figure 48) [187,188].

**Figure 49:**
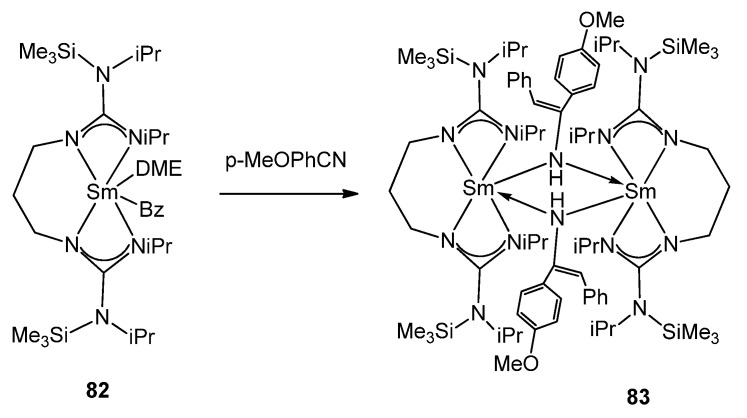
Nitrile insertion in alkyl samarium complex.

In contrast to the usual lack of reactivity observed in guanidinates of other metals, certain organolanthanides allow examples of the insertion of small unsaturated molecules into the metal–guanidinate bond. Complexes [(C_5_H_5_)_2_Ln(μ-η^1^:η^2^-N=C(NMe_2_)_2_)]_2_ (Ln = Y **79**, Gd **80**, Er **81**) can mono-insert phenyl isocyanate into the Ln–N bond to yield [(C_5_H_5_)_2_Ln(μ-η^1^:η^2^-OC(N=C(NMe_2_)_2_)NPh)]_2_ without further insertion or the usual isocyanate cyclotrimerization [110,189]. The presence of reactive alkyl groups, such as benzyl, offers the possibility to study alternative insertion processes [190,191,192]. Complex **82** inserts nitriles and isocyanates, generating binuclear complexes (Figure 49) [191].

**Figure 50:**
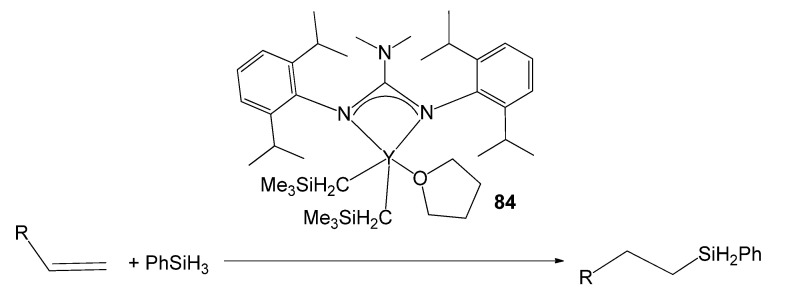
Alkene hydrosilylation with an yttrium guanidinato compound as catalyst.

A third route is the protonolysis reaction of alkyl derivatives with neutral guanidines [193]. In this way, an alkyl yttrium compound has been obtained with a guanidine with elevated steric demand and with the adequate control of the electronic deficiency of the metal. Compound **84** was highly efficient in the hydrosilylation of alkenes via hydride intermediates with excellent anti-Markovnikov selectivity (Figure 50) [193].

**Figure 51:**
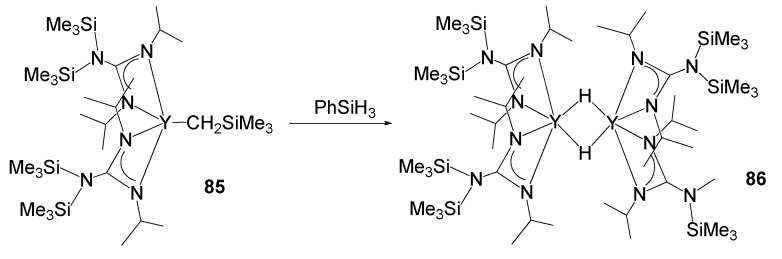
Synthesis of an yttrium hydride stabilized by guanidinato ligands.

The catalytic activity of rare-earth metal guanidinates also extends to olefin polymerization processes [194,195,196]. For example, the coordination of two guanidinate ligands allows the stabilization of hydride complexes. The appropriate steric bulk of the substituents on the nitrogen atoms provides high solubility of the lanthanide derivatives and enables Lewis base-free hydrides to be obtained from alkyl derivatives and silanes with an extremely low coordination index on the metal atom. Species such as compound **86** (Figure 51) have shown high catalytic activity in ethylene polymerization [194].

Such alkyl or hydride complexes stabilized by guanidinato ligands also allow ring-opening or polar monomer polymerizations [197,198].

To conclude this section, guanidinato ligands have met the needs for rare-earth metal molecular precursors for ALD or CVD techniques: the bidentate coordination mode allows to saturate coordinatively even large cations; the electronic delocalization across the ligand stabilizes the whole molecular structure; and the easy modification of the substituents allows to modulate the physical properties, all contributing to expand the library of potential precursors of new high quality materials obtained by these deposition techniques [199].

## 3. Conclusions

In conclusion, a great deal of work has been carried out in this field, but there is still scope for the development of new organometallic complexes, in which the choice of metal and the design of the ligands provide greater control of their chemical and physical properties. Guanidines provide easy access to such control, as they can be obtained by very straightforward and efficient syntheses from widely available reagents. The ease of formation of coordination and organometallic complexes, even in unconventional or unstable oxidation states, can be enhanced by potentially modifying these ligands to increase the number and type of donor atoms. A further step towards a firm foothold among the most successful ligands is the possibility to design and synthesize new chiral derivatives for enantioselective catalysis applications. So, again, an appropriate choice of precursors and methods to obtain these ligands, if possible, in a sustainable manner, opens up a whole range of possibilities for organometallic chemists.
